# Hydrate of Lime in Diarrhœa

**Published:** 1847-02

**Authors:** 


*Hydrate of Lime in Diarrhœa.*—Simple syrup, saturated with hydrate of lime, has been employed by Dr. Captaine as an antacid, in doses of from one scruple to half a drachm: and at the hospital Neckar, it has been given with much benefit instead of cow's milk in the diarrhœa of children.—*St. Louis Med. and Surg. Jour.*

